# A case of recurrent infective endocarditis after the commando procedure: echocardiographic diagnosis of a complex aorto-cavitary fistulization

**DOI:** 10.1093/ehjcr/ytaf096

**Published:** 2025-02-25

**Authors:** Tung-Yu Chen, Ming-Chon Hsiung, Ting-Chao Lin, Wein-Shung Kuo

**Affiliations:** Department of Anesthesiology, Cheng Hsin General Hospital, No. 45, Cheng Hsin St., Beitou District, Taipei 112401, Taiwan; Division of Cardiology, Cheng Hsin General Hospital, No. 45, Cheng Hsin St., Beitou District, Taipei 112401, Taiwan; Division of Cardiovascular Surgery, Heart Center, Cheng Hsin General Hospital, No. 45, Cheng Hsin St., Beitou District, Taipei 112401, Taiwan; Department of Anesthesiology, Cheng Hsin General Hospital, No. 45, Cheng Hsin St., Beitou District, Taipei 112401, Taiwan

## Case description

**Figure 1:**
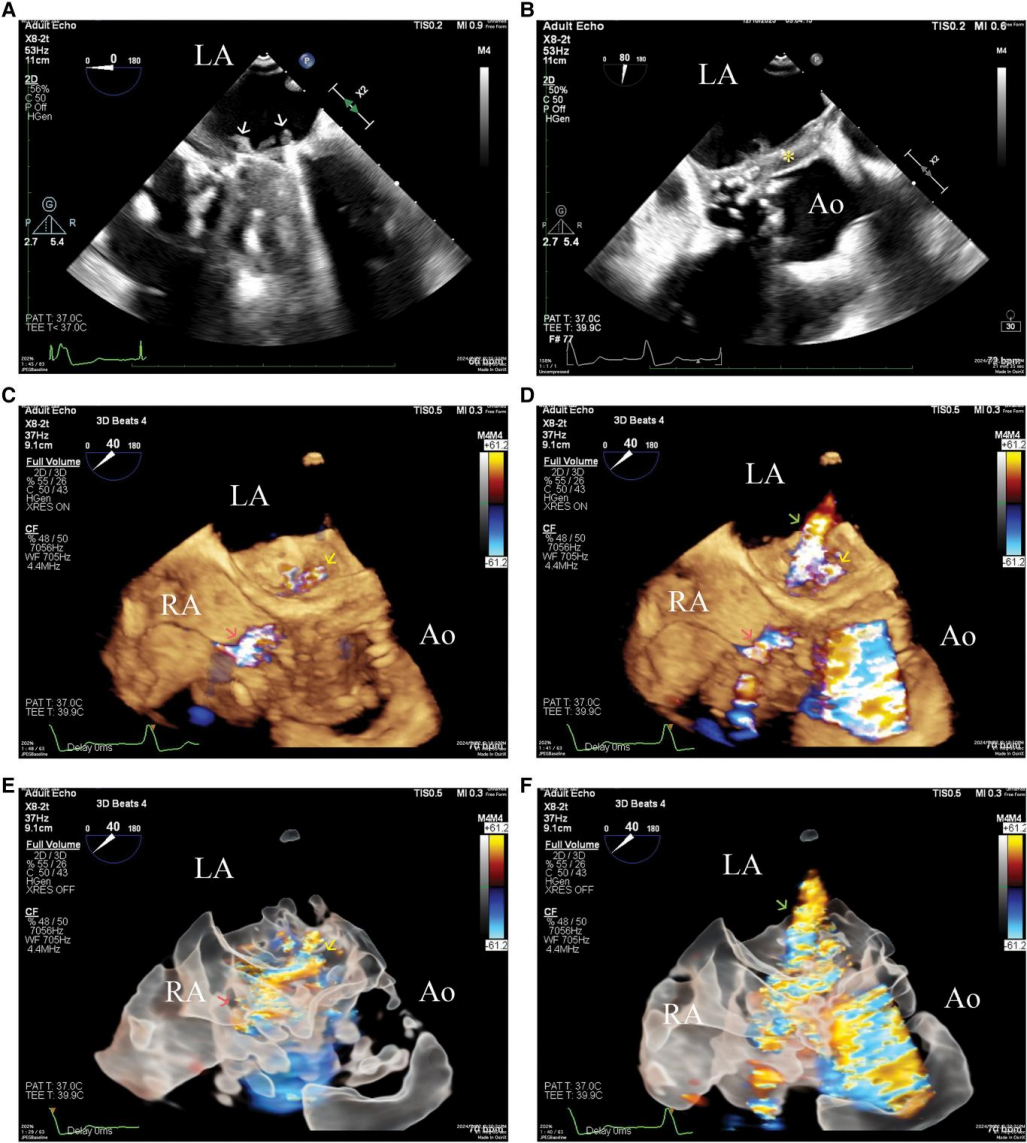
(*A*) 2D transoesophageal echocardiography showing vegetations (arrows) involving the prosthetic mitral valve in the four-chamber view. (*B*) 2D transoesophageal echocardiography showing echo-dense thickening (asterisk) of the aortic peri-valvular tissue. (*C*) 3D transoesophageal echocardiography with colour Doppler in the *diastolic phase* shows blood flow shunting of the aorto-cavitary fistulization into the left atrium (yellow arrow) and right atrium (red arrow). (*D*) 3D transoesophageal echocardiography with colour Doppler in the *systolic phase* shows a paravalvular leakage jet of the prosthetic mitral valve (green arrow), in addition to the blood flow shunting of the aorto-cavitary fistulization into the left atrium (yellow arrow) and right atrium (red arrow). (*E*) 3D glass view with colour Doppler in the *diastolic phase* delineates the continuity of the blood flow shunting into the left atrium (yellow arrow) and right atrium (red arrow). (*F*) 3D glass view with colour Doppler in the *systolic phase* shows a paravalvular leakage jet of the prosthetic mitral valve (green arrow), in addition to the aorto-cavitary fistulization.

A 51-year-old male with a history of first incident infective endocarditis underwent the commando procedure,^1^ where aortic and mitral valve replacement with mechanical prosthesis was performed, along with intervalvular fibrous body (IVFB) reconstruction. Seven months after the operation, the patient presented with low-grade fever and dyspnoea. Transthoracic echocardiography (TTE) was initially performed, which showed intracardiac shunting (see Supplementary material online, *Movie S1*); however, the full extent of the shunt was obscured by the shielding of the mechanical valves. Transoesophageal echocardiography (TEE) was performed for further evaluation, which showed severe valvular aortic regurgitation, with multiple vegetations attached to the prosthetic mitral valve (*Figure 1A*; Supplementary material online, *Movie S2*) and echo-dense thickening of the aortic peri-valvular tissue (*Figure 1B*). 3D TEE with colour Doppler revealed aorto-cavitary fistulization (ACF),^2^ with continuous blood flow shunting into the left atrium (LA) and right atrium (RA) (*Figure 1C* and *D*; Supplementary material online, *Movie S3*). 3D glass view delineates the continuity of the blood flow shunting into the LA and RA. An additional paravalvular leakage jet of the prosthetic mitral valve could also be seen during the systolic phase (*Figure 1E* and *F*; Supplementary material online, *Movie S4*). The blood culture grew *Staphylococcus epidermidis*. Empirical teicoplanin and ceftriaxone were used, which were then shifted to daptomycin according to sensitivity results. The patient was clinically stable without inotropic support. Under the impression of recurrent infective endocarditis, the decision for a redo Commando procedure was made. However, mere replacement of the aortic and mitral mechanical valves with reconstruction of the IVFB was not deemed sufficient, as the preoperative 3D TEE showed the fistula extending into the RA. The surgeon confirmed necrotic tissue around the prosthetic aortic valve, with the fistula extending into the RA through the membranous septum, which was excised and repaired with extension of the bovine patch used to reconstruct the IVFB. Temporary detachment of the septal tricuspid leaflet allowed full exposure of the membranous septum. Anteroseptal commissuroplasty was performed to avoid tricuspid regurgitation. Postoperative complete atrioventricular block, which has persisted since the first commando procedure, was managed with re-implantation of a permanent pacemaker. Follow-up TTE 7 months after the surgery showed well-functioning aortic and mitral mechanical valves, mild tricuspid regurgitation, and no residual intracardiac shunts (see Supplementary material online, *Movies S5* and *S6*).

We present a case of recurrent infective endocarditis after the commando procedure, where 3D TEE delineated ACF, a rare complication causing blood flow shunting and haemodynamic compromise.^3^ To our knowledge, this is the first case report showcasing the full extent of an ACF using the glass view of 3D TEE. Echocardiographic diagnosis of these types of fistulae is crucial, as extensive cardiac tissue destruction may involve even the membranous septum, which may necessitate surgical corrections beyond the standard commando procedure. The optimal characterization of the fistula tract guides surgical approaches, which ensures no residual fistula is left untreated.

## Supplementary Material

ytaf096_Supplementary_Data

## Data Availability

The authors confirm that the data supporting the findings of this study are available within the article and its Supplementary material.

## Supplementary material

**Consent:** Written informed consent was obtained from the patient for their information to be published in this article.

**Funding:** None declared.
